# TSC22D4 drives clear cell renal cell carcinoma progression and therapy resistance by stabilizing NRF2 through KEAP1 disruption

**DOI:** 10.1038/s41419-026-08950-4

**Published:** 2026-06-05

**Authors:** Bohan Zeng, Songbo Wang, Xuanzhi Zhang, Shancheng Ren, Chenji Wang, Dingwei Ye, Anbang Wang

**Affiliations:** 1 Department of Urology, Shanghai Changzheng Hospital, Naval Medical University, Shanghai, China; 2 Department of Urology, Fudan University Shanghai Cancer Center, Shanghai, China; 3 Department of Urology, First Affiliated Hospital of Nanjing Medical University, Nanjing, China; 4 State Key Laboratory of Genetic Engineering, MOE Engineering Research Center of Gene Technology, Shanghai Engineering Research Center of Industrial Microorganisms, School of Life Sciences, Fudan University, Shanghai, China

**Keywords:** Renal cell carcinoma, Renal cell carcinoma, Ubiquitylation

## Abstract

Clear cell renal cell carcinoma (ccRCC), the most common form of kidney cancer, is highly aggressive and difficult to manage due to its intrinsic resistance to oxidative stress. Nuclear factor erythroid 2-related factor 2 (NRF2), a master regulator of oxidative stress responses, is frequently overexpressed in ccRCC and is associated with poor prognosis. NRF2 promotes tumor progression by driving proliferation, metabolic reprogramming, and ferroptosis resistance; however, the upstream mechanisms controlling NRF2 stability remain incompletely understood. Here, we identify TSC22 domain family member 4 (TSC22D4) as a Kelch-like ECH-associated protein 1 (KEAP1)-interacting protein that directly binds to KEAP1 via a conserved ETGE motif. This interaction disrupts the KEAP1-NRF2 complex, preventing NRF2 ubiquitination and degradation, thereby enhancing NRF2 stability and activating antioxidant response element (ARE)-driven transcription. Notably, TSC22D4 upregulates solute carrier family 7 member 11 (SLC7A11), thereby suppressing ferroptosis and conferring resistance to sorafenib in ccRCC. TSC22D4 is markedly overexpressed in ccRCC and promotes tumor cell proliferation, invasion, and metastasis. Clinically, elevated TSC22D4 expression correlates with advanced disease stage and poor patient survival. In conclusion, TSC22D4 promotes ccRCC progression and sorafenib resistance by activating the KEAP1–NRF2–SLC7A11 axis and suppressing ferroptosis, highlighting TSC22D4 as a potential therapeutic target in ccRCC.

## Introduction

Renal cell carcinoma (RCC), which accounts for approximately 90% of kidney cancers, represents the most prevalent renal malignancy. Globally, approximately 434,419 new RCC cases and 155,702 deaths were reported in 2022 [1]. In the United States, 71,676 new cases and 15,259 deaths were recorded in the same year [2]. Oxidative stress plays a dual role in renal cancer initiation and progression. During tumor initiation, exogenous and endogenous oxidants induce chromosomal abnormalities and DNA mutations by directly damaging genetic material or disrupting repair mechanisms. Conversely, in established tumors, persistent oxidative stress activates antioxidant pathways that promote cancer progression and therapy resistance [3]. NRF2 is a transcription factor that regulates antioxidant response elements and plays significant roles in protection against oxidative and electrophilic stresses. Under normal conditions, NRF2 has been strictly regulated, while in several cancers, NRF2 is abnormally activated. Dysfunctional regulation, primarily through inactivation of KEAP1, is a major underlying mechanism.

KEAP1, a critical substrate adaptor and the primary negative regulator of NRF2, serves as the substrate-recognition component of the Cullin 3 (CUL3)–RING-box protein 1 (RBX1) E3 ubiquitin ligase complex. It directly interacts with NRF2 to facilitate its ubiquitination and subsequent proteasome-mediated degradation. The “hinge and latch” model posits that dimeric KEAP1 binds a single NRF2 molecule: one KEAP1 monomer engages the low-affinity “DLG” motif, while the other binds the high-affinity “ETGE” motif [4]. Under basal conditions, this complex remains stable, enabling constitutive ubiquitination and degradation of NRF2. However, modification of sensor cysteines in KEAP1 by oxidants or electrophiles induces conformational changes that disrupt NRF2 binding and impair KEAP1 function [5]. This allows newly stabilized NRF2 to accumulate, translocate to the nucleus, and drive the transactivation of target genes.

Beyond canonical regulation, the KEAP1-NRF2 axis is modulated by several proteins, including protein phosphatase 1 regulatory subunit 13-like (IASPP) [6], dipeptidyl peptidase 3 (DPP3) [7], dipeptidyl peptidase 9 (DPP9) [8], and HECT domain and ankyrin repeat-containing E3 ubiquitin protein ligase 1 (HACE1) [9]. These proteins contain a conserved “ETGE” or “DLG” sequence that enables them to compete with NRF2 for binding to KEAP1, thereby preventing nascent NRF2 from degradation. Upon nuclear translocation, NRF2 forms heterodimers with small MAF proteins (MAF bZIP transcription factors). This complex binds to antioxidant response elements (ARE), activating transcription of hundreds of target genes. Notably, NRF2 directly regulates key ferroptosis-related genes, including SLC7A11 [10], GPX4 [11], establishing a critical link between NRF2 signaling and ferroptosis resistance.

Given the complex regulation of the KEAP1-NRF2 axis, we next investigated potential novel regulators of this pathway. Based on our preliminary findings and mass spectrometry data from this study, we selected TSC22D4 as the focus of further investigation [12, 13]. The TGF-β-stimulated clone 22 domain (TSC22D) family comprises four members (TSC22D1-TSC22D4). TSC22D4 plays established roles in cellular metabolism, demonstrated by its capacity to prevent and reverse hyperglycemia, glucose intolerance, and insulin resistance in rodent models [14]. Conversely, loss-of-function studies reveal that TSC22D4 deficiency impairs the TCA cycle, mitochondrial integrity, and triglyceride metabolism in hepatocytes, thereby promoting non-alcoholic steatohepatitis [15]. Clinically, elevated TSC22D4 expression serves as a molecular signature of cancer cachexia, with higher levels correlating with cachexia severity [16]. Regarding cellular functions, TSC22D4 is initially characterized as a tumor suppressor: TSC22D4-TSC22D1 heterodimers facilitate cell-cycle exit in medulloblastoma [17] and suppress cellular senescence in esophageal carcinoma [18]. However, its role in renal cell carcinoma, particularly in the context of oxidative stress and NRF2 signaling, remains unclear. In this study, our study identifies a novel oncogenic role for TSC22D4 in ccRCC. We demonstrate that TSC22D4 activates the NRF2 pathway by disrupting KEAP1-mediated degradation, consequently promoting tumor progression.

Importantly, NRF2 has been increasingly recognized as a key regulator of ferroptosis. Ferroptosis is a regulated form of cell death characterized by iron-dependent lipid peroxidation of polyunsaturated fatty acid-containing phospholipids. Accumulation of lipid hydroperoxides disrupts membrane integrity and ultimately leads to cell death. The sensitivity of cells to ferroptosis is tightly controlled by metabolic pathways involved in iron homeostasis, lipid metabolism, and redox regulation. Among these, the glutathione peroxidase 4 (GPX4)-glutathione axis represents a central defense system that detoxifies lipid hydroperoxides and prevents ferroptosis [19]. In parallel, cystine uptake through the system xc− antiporter sustains glutathione synthesis and maintains cellular redox balance [20]. Besides, the ferroptosis suppressor protein 1 (FSP1)–coenzyme Q10 (CoQ10) pathway functions as a nicotinamide adenine dinucleotide phosphate (NADPH)-dependent ubiquinone reductase system that generates the lipophilic antioxidant ubiquinol to terminate lipid peroxidation chain reactions [21, 22]. Meanwhile, the GTP cyclohydrolase 1 (GCH1)–tetrahydrobiopterin (BH4) axis provides another ferroptosis-protective mechanism through the synthesis of tetrahydrobiopterin, a potent radical-trapping antioxidant that limits phospholipid peroxidation [23]. Dysregulation of these antioxidant pathways enables tumor cells to suppress ferroptosis and survive under oxidative stress conditions. Consequently, ferroptosis resistance has emerged as an important mechanism contributing to tumor progression and therapeutic resistance. Therefore, as a regulator of NRF2, we investigated whether TSC22D4 can regulate ferroptosis in tumor cells and consequently induce therapy resistance.

## Results

### TSC22D4 interacts with KEAP1 through the ETGE motif

**Fig. 1:**
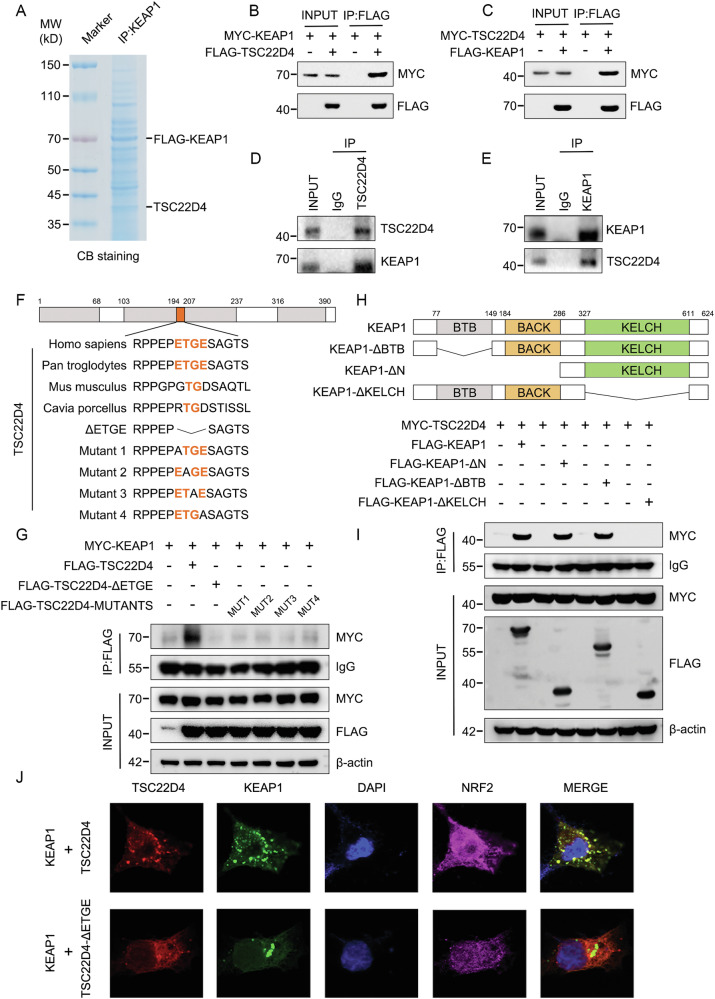
TSC22D4 interacts with KEAP1 through a conserved ETGE motif. **A** KEAP1-associated proteins were isolated from 293 T cells stably expressing FLAG-KEAP1 by immunoprecipitation, separated by SDS-PAGE, and visualized by Coomassie Brilliant Blue staining. Bands corresponding to FLAG-KEAP1 and TSC22D4 are indicated. **B, C** Reciprocal exogenous co-immunoprecipitation (Co-IP) assays showing the interaction between KEAP1 and TSC22D4 in 293 T cells. FLAG-TSC22D4 was immunoprecipitated and MYC-KEAP1 was detected (**B**). FLAG-KEAP1 was immunoprecipitated and MYC-TSC22D4 was detected (**C**). Western blotting was performed to detect the indicated proteins. **D, E** Co-IP assays demonstrating the interaction between endogenous KEAP1 and endogenous TSC22D4 in 293 T cells. TSC22D4 or KEAP1 was immunoprecipitated, and the indicated interacting proteins were detected by Western blotting. **F** Schematic representation of wild-type TSC22D4 and ETGE-site mutants (ΔETGE and four-point mutants: mutant 1- mutant 4). Conserved ETGE sequences across species are aligned. **G** Co-IP assays assessing the interaction between MYC-KEAP1 and FLAG-tagged TSC22D4 WT, ΔETGE, and ETGE point mutants. **H** Schematic diagram of wild-type KEAP1 and domain-deleted KEAP1 mutants: KEAP1-ΔBTB, KEAP1-ΔN, and KEAP1-ΔKELCH. **I** Co-IP assays evaluating the interaction between MYC-TSC22D4 and FLAG-tagged KEAP1 WT or truncation mutants. **J** Confocal immunofluorescence images showing the subcellular localization and colocalization of TSC22D4 and KEAP1. KEAP1, TSC22D4, NRF2, and DAPI signals are shown for cells expressing TSC22D4-WT (top row) or TSC22D4-ΔETGE (bottom row). Scale bars are indicated.

To identify KEAP1-interacting proteins, we established a stable 293 T cell line expressing FLAG-tagged KEAP1 and performed co-immunoprecipitation (Co-IP) to isolate putative binding partners (Fig. 1A). Mass spectrometry-based proteomic analysis of the Co-IP products revealed that TSC22D4 specifically associates with KEAP1 (Fig. S1A). Exogenous and endogenous Co-IP experiments confirmed the interaction between TSC22D4 and KEAP1 (Fig. 1B–E). Because KEAP1 typically recognizes substrates through ETGE motifs, we analyzed the amino acid sequence of TSC22D4 and identified a conserved ETGE motif. We therefore generated ETGE-motif mutants by deleting the entire motif or substituting individual residues within the motif (Fig. 1F). Mutation of this motif abolished the interaction between TSC22D4 and KEAP1. Moreover, Co-IP assays using KEAP1 truncation mutants (KEAP1-ΔBTB, KEAP1-ΔN, and KEAP1-ΔKELCH) demonstrated that TSC22D4 binding requires the KELCH domain of KEAP1, as KEAP1-ΔKELCH failed to precipitate TSC22D4 (Fig. 1G–I). Confocal immunofluorescence microscopy further demonstrated that KEAP1 and TSC22D4 were spatially co-localized within cells. Collectively, these findings identify TSC22D4 as a KEAP1-binding protein that interacts with KEAP1 through an ETGE motif-dependent mechanism.

### TSC22D4 attenuates the ubiquitin-proteasome degradation of NRF2

**Fig. 2:**
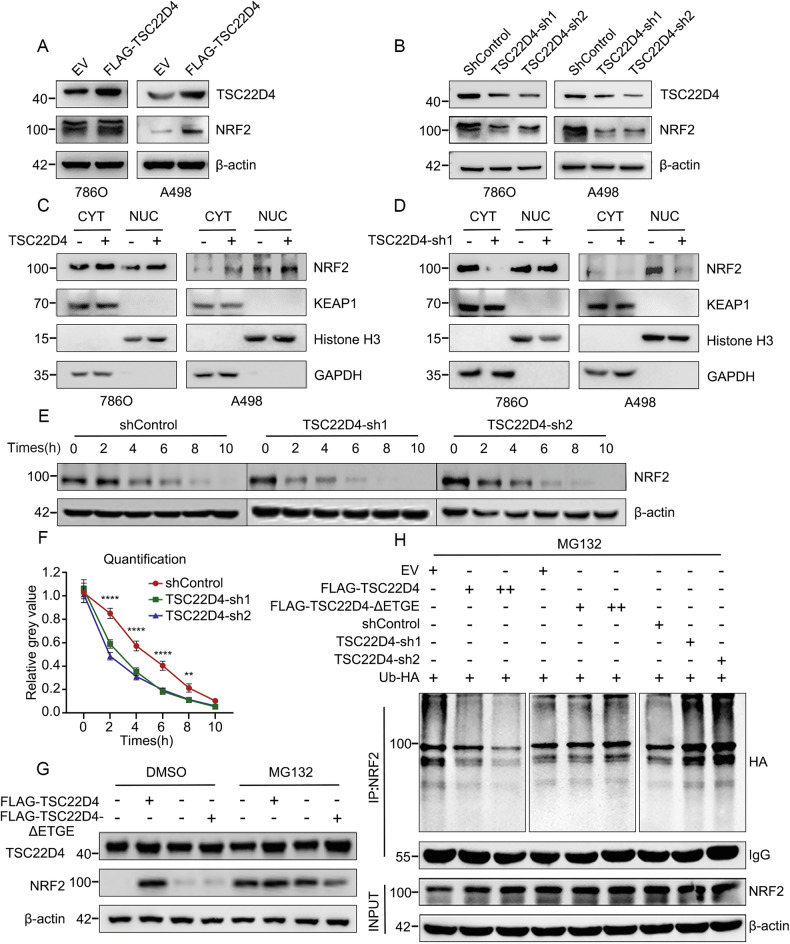
TSC22D4 stabilizes NRF2 by suppressing ubiquitin-mediated proteasomal degradation. **A**, **B** Western blot analysis of NRF2 levels in 786-O and A498 cells following TSC22D4 overexpression (**A**) or knockdown using shTSC22D4 (**B**). **C**, **D** β-actin served as a loading control. Nuclear and cytoplasmic fractionation analysis of NRF2 and KEAP1 in 786-O and A498 cells with TSC22D4 overexpression (**C**) or TSC22D4 knockdown (**D**). GAPDH and Histone H3 were used as cytoplasmic and nuclear markers, respectively. **E**, **F** Cycloheximide (CHX; 100 μg/mL) chase assay assessing NRF2 protein stability in 786-O cells expressing shControl or shTSC22D4 (sh1 or sh2). NRF2 degradation was monitored from 0 to 10 h (**E**), and signal intensities were quantified by densitometry (**F**). **G** Western blot analysis of NRF2 levels in 786-O cells expressing empty vector, FLAG-TSC22D4 WT, or FLAG-TSC22D4-ΔETGE following treatment with MG132 (20 μM, 6 h) or DMSO. β-actin served as a loading control. **H** Ubiquitination of NRF2 assessed by immunoprecipitation of endogenous NRF2 in 293 T cells transfected with empty vector, TSC22D4 WT, TSC22D4-ΔETGE, shControl, shTSC22D4 (sh1 or sh2), and HA-ubiquitin. Cells were incubated with MG132 (1 μM) for 24 h before harvest. Ubiquitinated NRF2 was detected by immunoprecipitation of NRF2 and followed by anti-HA immunoblotting. Input protein levels were verified by Western blot.

KEAP1 is a key E3 ubiquitin ligase that orchestrates the ubiquitination and proteasomal degradation of NRF2. Given the interaction between TSC22D4 and KEAP1, we hypothesized that TSC22D4 may modulate NRF2 stability. To test this hypothesis, we assessed NRF2 expression after modulating of TSC22D4 in 786-O and A498 renal carcinoma cells (Fig. 2A, B). Overexpression of TSC22D4 increased NRF2 protein abundance, whereas TSC22D4 silencing decreased NRF2 levels. Because NRF2 stabilization is commonly associated with enhanced nuclear accumulation, we performed nuclear and cytoplasmic fractionation and examined NRF2 protein levels in each fraction. NRF2 protein levels in both the cytoplasmic and nuclear fractions were positively associated with TSC22D4 expression (Fig. 2C, D). To further investigate the underlying mechanism, we monitored NRF2 protein stability using cycloheximide (CHX) chase assays and observed accelerated NRF2 degradation upon TSC22D4 depletion (Fig. 2E, F). This effect was reversed by the proteasome inhibitor MG132, indicating the involvement of the ubiquitin-proteasome pathway (Fig. 2G). Consistently, TSC22D4 overexpression markedly reduced NRF2 ubiquitination, whereas the ETGE-mutant TSC22D4 failed to exert this effect. Conversely, TSC22D4 knockdown increased NRF2 ubiquitination (Fig. 2H). Collectively, these findings indicate that TSC22D4 stabilizes NRF2 by suppressing its ubiquitination and proteasomal degradation.

### TSC22D4 activates the NRF2 antioxidant program in ccRCC

**Fig. 3:**
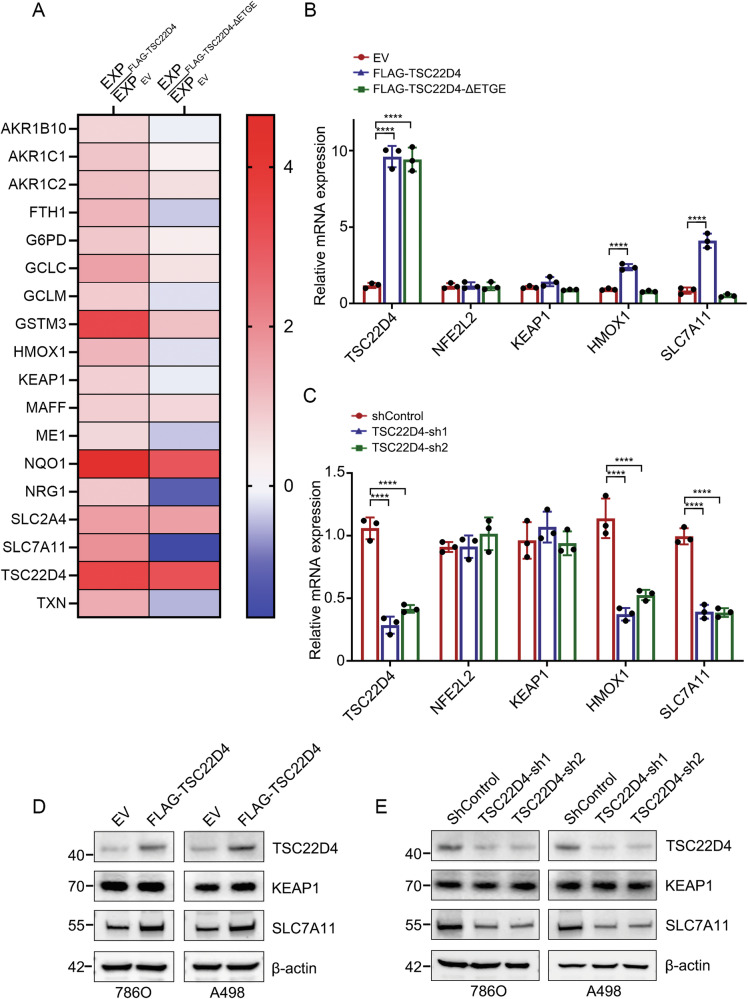
TSC22D4 promotes the expression of NRF2 target genes in ccRCC. **A** Heatmap showing the relative expression of NRF2 downstream genes in 786-O cells transfected with empty vector (EV), FLAG-TSC22D4, or FLAG-TSC22D4-ΔETGE. Differentially expressed genes between TSC22D4-overexpressing cells and control cells are shown. **B**, **C** Quantitative RT-PCR analysis of TSC22D4, NFE2L2, KEAP1, HMOX1, and SLC7A11 mRNA levels in 786-O cells following TSC22D4 overexpression with empty vector, FLAG-TSC22D4 or FLAG-TSC22D4-ΔETGE (**B**) or knockdown using shTSC22D4 (sh1 or sh2) (**C**). **D**, **E** Western blot analysis of TSC22D4, KEAP1, SLC7A11, and β-actin in 786-O and A498 cells with TSC22D4 overexpression (**D**) or TSC22D4 knockdown (**E**). β-actin served as a loading control. Data represent mean ± SD from three independent experiments. ns, not significant; *p < 0.05, **p < 0.01, ***p < 0.001, ****p < 0.0001 vs. control group (Student’s *t* test).

To further assess the impact of TSC22D4 on NRF2 downstream signaling, we generated ccRCC cell lines stably expressing either wild-type TSC22D4 or the ETGE-motif mutant. RT-qPCR analysis showed that wild-type TSC22D4 significantly upregulated canonical NRF2 target genes, including *HMOX1 (heme oxygenase 1), GCLC (glutamate-cysteine ligase catalytic subunit)*, and *GCLM (glutamate-cysteine ligase modifier subunit)* (Fig. 3A). Notably, *SLC7A11* expression was also markedly elevated, suggesting a potential role in ferroptosis regulation. By contrast, the ETGE-motif mutant showed a reduced ability to activate these genes. Consistently, TSC22D4 knockdown led to their coordinated suppression (Fig. 3B, C). Western blot analysis further confirmed corresponding changes at the protein level (Fig. 3D, E). In conclusion, these findings demonstrate that TSC22D4 activates the NRF2 downstream transcriptional signaling in ccRCC cells.

### TSC22D4 promotes proliferation, migration and invasion in clear cell renal carcinoma cells

**Fig. 4:**
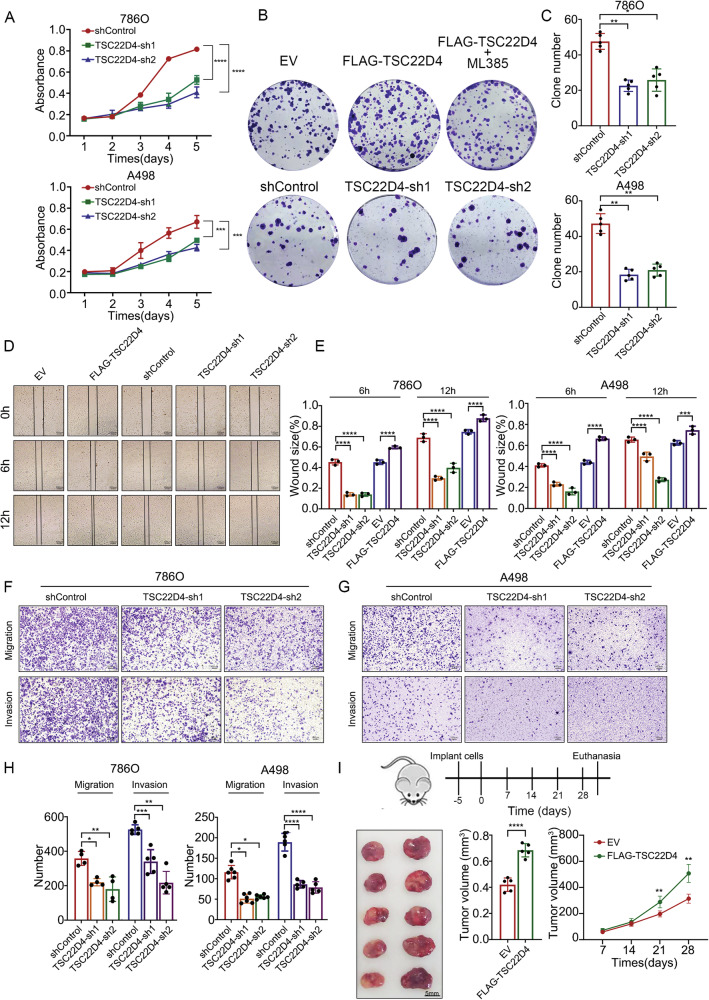
TSC22D4 promotes cell proliferation, migration, invasion, and tumor growth in ccRCC. **A** Cell proliferation of 786-O and A498 cells expressing shControl or shTSC22D4 (sh1 or sh2) was assessed using the CCK-8 assay. Data represent mean ± SD from three independent experiments, each performed with six replicates (*n* = 6). **B** Representative images of colony formation assays in 786-O and A498 cells stably expressing empty vector (EV), FLAG-TSC22D4, ML385-treated FLAG-TSC22D4 cells (5 μM, 48 h), shControl, TSC22D4-sh1, or TSC22D4-sh2. **C** Quantification of colony numbers from 786-O and A498 cells expressing shControl, TSC22D4-sh1, or TSC22D4-sh2. Data represent mean ± SD from three independent experiments, each with five replicates (*n* = 5). **D**, **E** Wound-healing assays were performed in 786-O and A498 cells with the indicated treatments. Representative images at 0 h, 6 h, and 12 h (**D**) and quantification of wound closure (**E**) are shown. Data represent mean ± SD from three independent experiments (*n* = 3). **F**–**H** Migration and invasion assays of 786-O (**F**) and A498 (**G**) cells expressing shControl, TSC22D4-sh1, or TSC22D4-sh2 using Transwell chambers. Quantification of migrated and invaded cells is shown in (**H**). Data represent mean ± SD from at least five replicates (*n* = 5). **I** Subcutaneous xenograft assay using 786-O cells stably expressing EV or FLAG-TSC22D4 to evaluate in vivo tumor growth. Schematic diagram, representative tumor images, tumor volumes, and tumor growth curves over 28 days are shown. ns, not significant; *P < 0.05, **P < 0.01, ***P < 0.001, ****P < 0.0001 vs. control (Student’s *t* test).

To delineate the functional role of TSC22D4 in ccRCC, we performed a series of cellular assays. CCK-8 assays revealed that TSC22D4 knockdown significantly inhibited proliferation in both 786-O and A498 cells (Fig. 4A). Consistently, clonogenic assays showed markedly reduced colony formation following TSC22D4 depletion, whereas the TSC22D4-driven clonogenic growth was abolished by the NRF2 inhibitor ML385, indicating an NRF2-dependent mechanism (Fig. 4B, C). In wound-healing assays, TSC22D4 overexpression accelerated cell migration, whereas TSC22D4 silencing impaired migratory capacity (Fig. 4D, E). Transwell migration and invasion assays further corroborated these results, demonstrating significantly reduced migratory and invasive abilities in TSC22D4-depleted cells (Fig. 4F–H). In vivo, subcutaneous xenograft assays showed that tumors derived from TSC22D4-overexpressing cells were substantially larger and exhibited accelerated growth compared with control tumors (Fig. 4I). Collectively, these findings demonstrate that TSC22D4 enhances ccRCC cell proliferation, migration, and invasion in vitro and promotes tumor growth in vivo.

### TSC22D4 confers resistance to ferroptosis in ccRCC

**Fig. 5:**
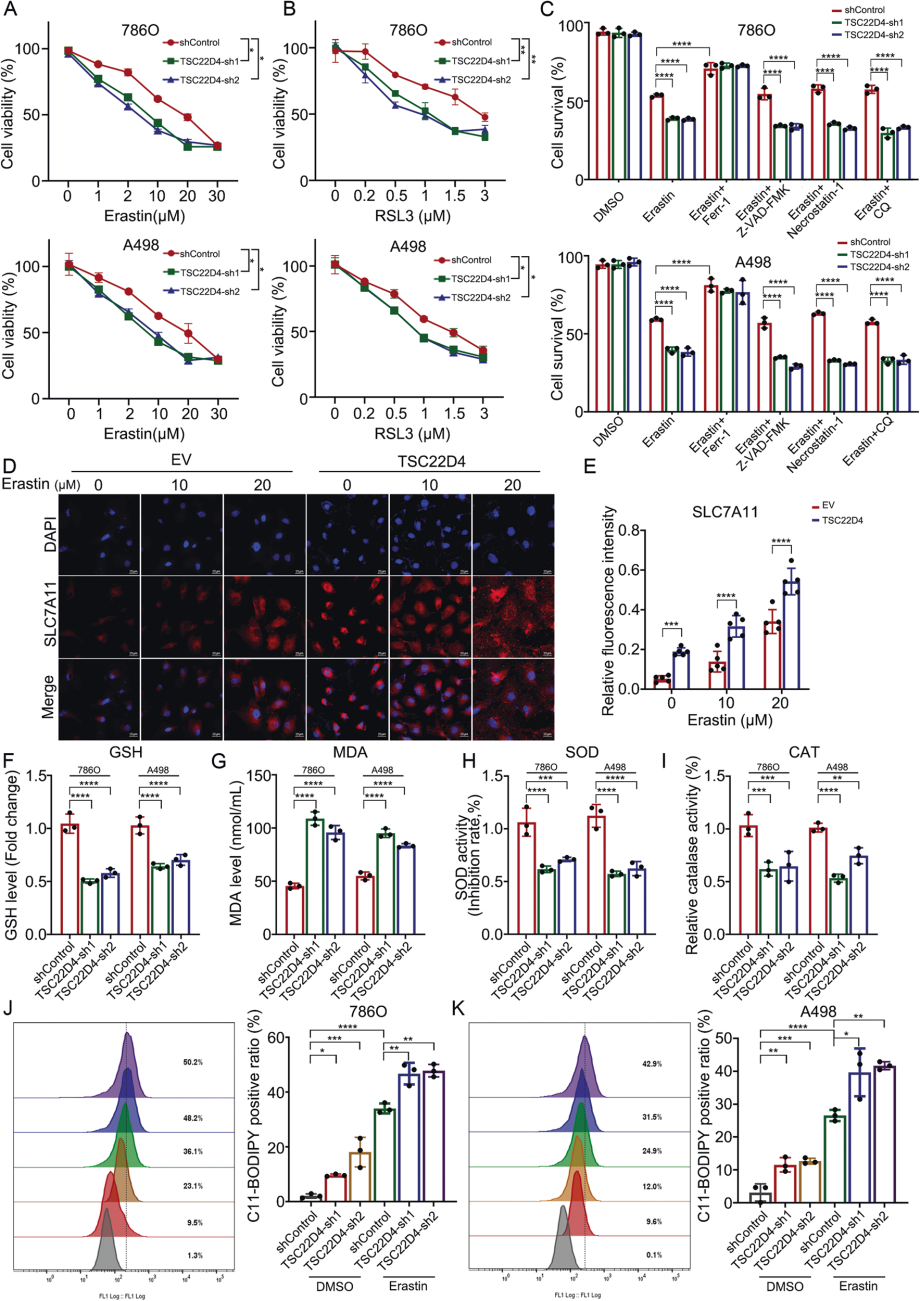
TSC22D4 regulates ferroptosis in ccRCC. **A**, **B** Cell viability of 786-O and A498 cells stably expressing shControl, TSC22D4-sh1, or TSC22D4-sh2 following treatment with erastin (**A**) or RSL3 (**B**). Viability was determined using the CCK-8 assay. **C** Rescue experiments showing the effects of erastin (2 µM), ferrostatin-1 (Fer-1, 10 µM), Z-VAD-FMK (40 µM), necrostatin-1 (10 µM), and chloroquine (CQ, 1 µM) on cell survival in 786-O and A498 cells expressing shControl or shTSC22D4. Cell survival was measured by CCK-8 assay. **D**, **E** Immunofluorescence analysis of SLC7A11 in 786-O cells expressing EV or TSC22D4 following treatment with erastin (0, 10, or 20 μM). Representative fluorescence images (**D**) and quantification of relative SLC7A11 fluorescence intensity (**E**) are shown. **F**–**I** Measurement of intracellular redox parameters in 786-O and A498 cells expressing shControl, TSC22D4-sh1, or TSC22D4-sh2, including glutathione (GSH) levels (**F**), malondialdehyde (MDA) levels (**G**), superoxide dismutase (SOD) activity (**H**), and catalase (CAT) activity (**I**). **J**, **K** Flow cytometric analysis of lipid peroxidation using C11-BODIPY in 786-O (**J**) and A498 (**K**) cells expressing shControl, TSC22D4-sh1, or TSC22D4-sh2 following DMSO or erastin treatment. Representative histograms and quantification of C11-BODIPY-positive cells are shown. Data represent mean ± SD from three independent experiments. ns, not significant; *P < 0.05, **P < 0.01, ***P < 0.001, ****P < 0.0001 vs. control (Student’s *t* test).

Given our observation that TSC22D4 markedly upregulates SLC7A11, a central regulator of ferroptosis, we hypothesized that TSC22D4 may confer ferroptosis resistance in renal cell carcinoma. To test this, we treated cells with the ferroptosis inducers erastin and RSL3 and found that TSC22D4 knockdown sensitized both 786-O and A498 cells to ferroptosis (Fig. 5A, B). To exclude the involvement of alternative cell death pathways, cells were co-treated with erastin and pathway-specific inhibitors, including ferrostatin-1 (Fer-1), Z-VAD-FMK, necrostatin-1, and chloroquine (CQ). Only Fer-1 restored cell viability, indicating that the enhanced cell death caused by TSC22D4 depletion was mainly attributable to ferroptosis (Fig. 5C). Immunofluorescence analysis showed that TSC22D4 overexpression enhanced SLC7A11 signals under basal conditions and after erastin treatment, with stronger signals observed at higher erastin concentrations (Fig. 5D, E). Furthermore, TSC22D4 knockdown reduced intracellular reduced glutathione (GSH), superoxide dismutase (SOD), and catalase (CAT) levels, while increasing malondialdehyde (MDA) levels in both cell lines, consistent with impaired redox homeostasis and enhanced lipid peroxidation (Fig. 5F–I). C11-BODIPY flow cytometry further showed that TSC22D4 knockdown increased lipid peroxidation under both basal and erastin-treated conditions, with a more pronounced effect after erastin exposure (Fig. 5J, K). Together, these findings demonstrate that TSC22D4 protects ccRCC cells from ferroptosis by maintaining redox homeostasis and promoting SLC7A11 expression.

### TSC22D4 confers sorafenib resistance by upregulation of SLC7A11 in ccRCC cells

**Fig. 6:**
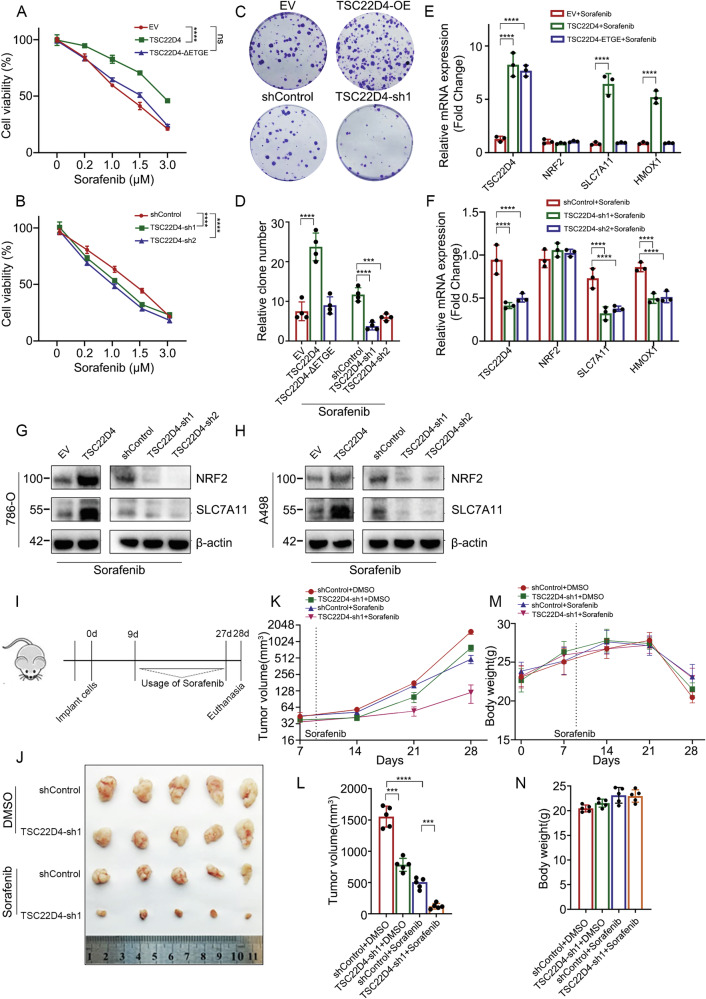
TSC22D4 enhances resistance to sorafenib by upregulating SLC7A11 in ccRCC. **A**, **B** Cell viability of 786-O and A498 cells expressing empty vector (EV), TSC22D4, TSC22D4-ΔETGE, shControl, TSC22D4-sh1, or TSC22D4-sh2 following sorafenib treatment at the indicated concentrations (0-3 μM). Viability was measured using the CCK-8 assay. **C**, **D** Representative colony formation images (**C**) and quantitative analysis (**D**) of 786-O cells expressing EV, TSC22D4, TSC22D4-ΔETGE, shControl, TSC22D4-sh1, or TSC22D4-sh2 under sorafenib treatment. Data represent mean ± SD from four replicates (*n* = 4). **E**, **F** qRT-PCR analysis of TSC22D4, NRF2, SLC7A11, and HMOX1 mRNA expression in 786-O cells expressing EV, TSC22D4, TSC22D4-ΔETGE (**E**) or shControl, TSC22D4-sh1, TSC22D4-sh2 (**F**) after treatment with sorafenib (10 μM). ACTB served as an internal reference (*n* = 3). **G**, **H** Western blot analysis of NRF2, SLC7A11, and β-actin in 786-O (**G**) and A498 (**H**) cells expressing EV, TSC22D4, shControl, TSC22D4-sh1, or TSC22D4-sh2 following sorafenib treatment (10 μM). **I** Schematic diagram of the mouse xenograft experimental design. 786-O cells stably expressing shControl or TSC22D4-sh1 were injected subcutaneously (1 × 10⁶ cells/mouse). Mice received daily DMSO or sorafenib (10 mg/kg) for 18 days. **J** Representative tumor images collected at the experimental endpoint. **K**, **L** Tumor growth curves (**K**) and tumor volume quantification (**L**) from xenografted mice treated with DMSO or sorafenib (*n* = 5). **M**, **N** Body-weight changes (**M**) and final body weight measurements (**N**) of mice during the treatment period (*n* = 5). Data represent mean ± SD. ns, not significant; *P < 0.05, **P < 0.01, ***P < 0.001, ****P < 0.0001 vs. control (Student’s *t* test).

Sorafenib, a multi-target tyrosine kinase inhibitor used for advanced renal carcinoma therapy, has been mechanistically linked to ferroptosis induction. We therefore examined whether TSC22D4 contributes to sorafenib resistance in ccRCC cells. Using 786-O cells overexpressing wild-type or ETGE-mutant TSC22D4, we found that wild-type TSC22D4 markedly increased resistance to sorafenib, whereas the ETGE-mutant form failed to confer protection (Fig. 6A, B). Clonogenic assays further demonstrated that TSC22D4 knockdown significantly impaired colony-forming capacity under sorafenib treatment (Fig. 6C, D). We next assessed whether sorafenib modulates the TSC22D4-SLC7A11 regulatory axis and found that TSC22D4 knockdown reduced SLC7A11 mRNA and protein expression in the presence of sorafenib (Fig. 6E–H). In vivo, TSC22D4 knockdown in a nude mouse xenograft model decreased tumor growth, and this effect was further potentiated by sorafenib administration (Fig. 6I, J). To further validate these findings in a clinically relevant model, we established patient-derived renal cancer organoids from fresh tumor tissues and introduced lentiviral-mediated TSC22D4 knockdown. Notably, under sorafenib treatment, TSC22D4-silenced organoids exhibited significantly reduced growth compared with control organoids, indicating that TSC22D4 depletion enhances sensitivity to sorafenib (Fig. S1E, F). Collectively, these findings indicate that TSC22D4 promotes sorafenib resistance in ccRCC by sustaining SLC7A11 expression and attenuating ferroptosis.

### TSC22D4 expression is related to the prognosis in ccRCC patients

**Fig. 7:**
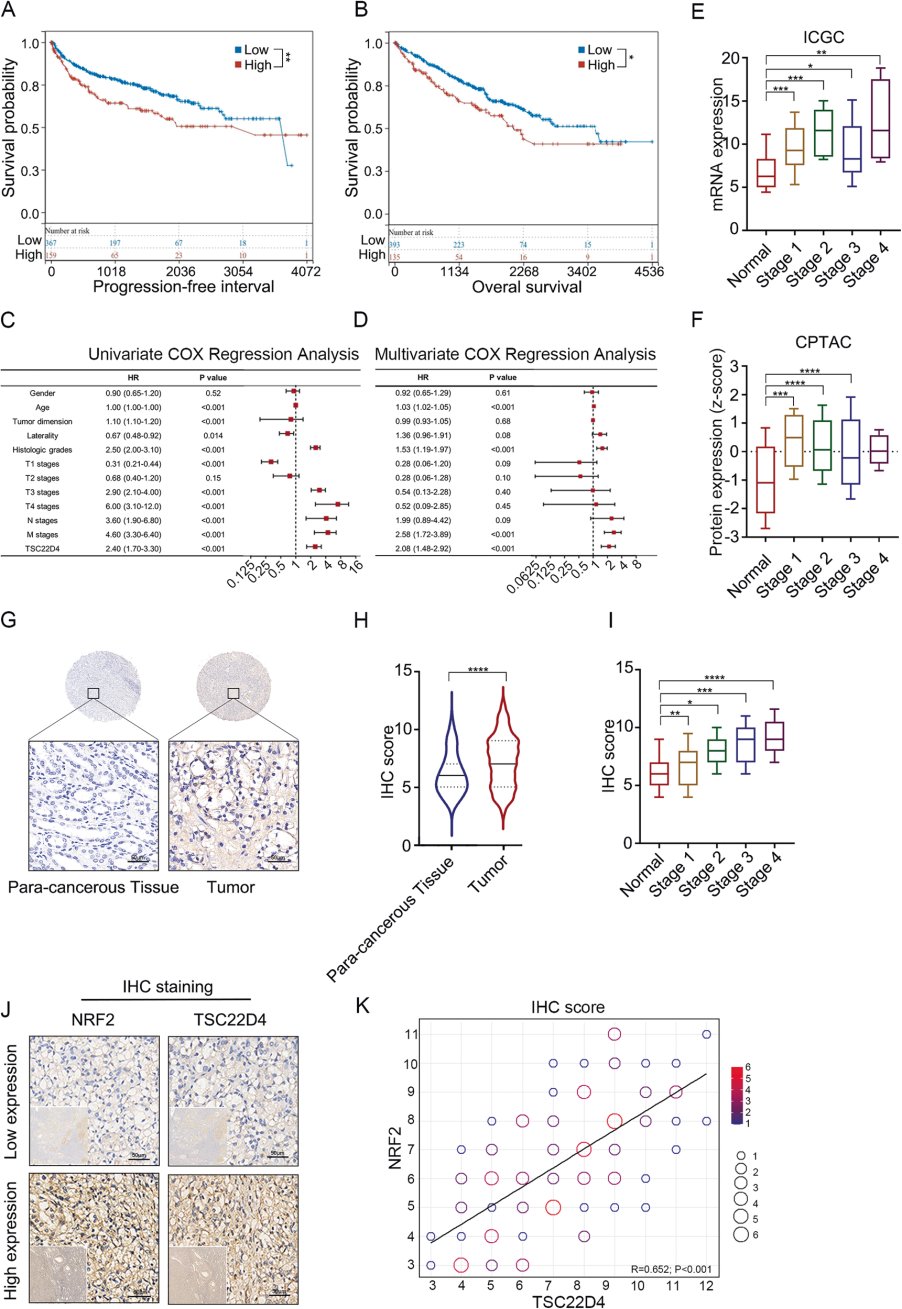
TSC22D4 expression correlates with disease progression and poor prognosis in ccRCC patients. **A**, **B** Kaplan-Meier survival curves for progression-free interval (**A**) and overall survival (**B**) in the TCGA-KIRC cohort stratified by TSC22D4 expression. Log-rank test: *P* < 0.001; HR = 1.54 (1.12–2.13) for PFI, and *P* < 0.05; HR = 1.49 (1.07–2.06) for OS. **C**, **D** Univariate (**C**) and multivariate (**D**) Cox proportional hazards regression analyses evaluating TSC22D4 expression and clinicopathological variables in ccRCC patients. Hazard ratios (HR) with 95% confidence intervals (CI) are shown. **E** TSC22D4 mRNA expression across American Joint Committee on Cancer (AJCC) stages in the ICGC cohort. **F** TSC22D4 protein abundance (Z-score) across AJCC stages in the CPTAC cohort. **G**–**I** Representative immunohistochemistry (IHC) images of TSC22D4 in matched paracancerous and tumor tissues from the FUSCC cohort (*n* = 101) (**G**); violin plot of TSC22D4 IHC scores comparing paracancerous vs. tumor tissues (**H**); and TSC22D4 IHC scores across AJCC stages (**I**). **J**, **K** Representative IHC staining images of NRF2 and TSC22D4 in ccRCC tissues categorized by low and high expression (**J**). Correlation analysis of NRF2 and TSC22D4 IHC scores using Pearson’s correlation (**K**). R = 0.652, *P* < 0.001. ns, not significant; *P < 0.05, **P < 0.01, ***P < 0.001, ****P < 0.0001 vs. control (Student’s *t* test).

**Fig. 8:**
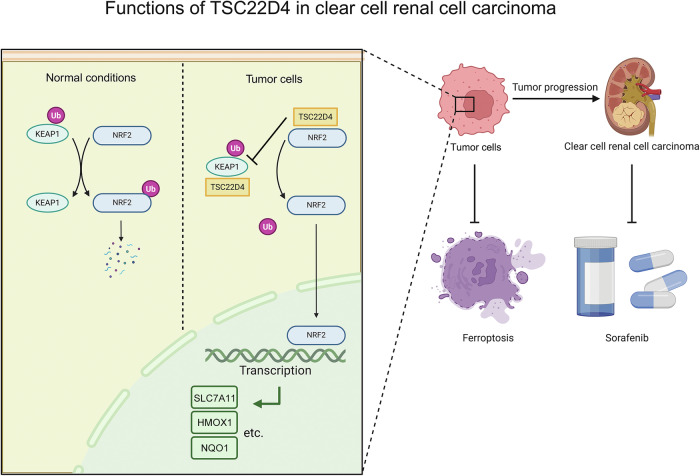
Proposed model of TSC22D4-mediated regulation of NRF2 signaling in ccRCC. Under physiological conditions, KEAP1 binds NRF2 and promotes its ubiquitination and proteasomal degradation. In ccRCC, TSC22D4 binds KEAP1 through its conserved ETGE motif and interferes with KEAP1-mediated NRF2 ubiquitination. Stabilized NRF2 accumulates in the nucleus and activates antioxidant response element (ARE)-driven transcription, including the induction of NRF2 target genes such as SLC7A11, HMOX1, and NQO1, thereby promoting tumor cell survival and progression. TSC22D4 also enhances ferroptosis resistance and sorafenib resistance by upregulating SLC7A11 expression in ccRCC.

To evaluate the prognostic relevance of TSC22D4 in ccRCC, we analyzed multiple public datasets. In the Cancer Genome Atlas (TCGA) cohort, elevated TSC22D4 expression was significantly associated with shorter progression-free interval (PFI) and overall survival (OS), indicating worse clinical outcomes (Fig. 7A, B). Univariate and multivariate Cox regression analyses further identified TSC22D4 as an independent prognostic factor (Fig. 7C, D). Analysis of the International Cancer Genome Consortium (ICGC) dataset showed that TSC22D4 expression was significantly higher in tumor tissues than in adjacent normal tissues and increased progressively with tumor stage (Fig. 7E) [24]. Similar trends were observed at the protein level in the Clinical Proteomic Tumor Analysis Consortium (CPTAC) proteomic dataset (Fig. 7F) [25]. Consistent with these findings, immunohistochemical analysis of the Fudan University Shanghai Cancer Center (FUSCC) cohort confirmed significantly higher TSC22D4 expression in tumor tissues than in adjacent normal tissues (Fig. 7G–I). Moreover, IHC score analysis revealed a positive correlation between TSC22D4 and NRF2 expression, supporting a functional association between the two proteins in clinical samples (Fig. 7J, K). In summary, TSC22D4 promotes ccRCC progression, ferroptosis resistance, and sorafenib resistance by competitively binding KEAP1 through its conserved ETGE motif, thereby stabilizing NRF2 and activating ARE-driven target genes, particularly SLC7A11 (Fig. 8).

## Discussion

Clear cell renal cell carcinoma remains a globally prevalent malignancy with persistently high incidence and mortality. Advanced ccRCC is largely incurable with current therapeutic strategies, resulting in poor clinical outcomes. Conventional modalities such as chemotherapy and radiotherapy exert antitumor effects through reactive oxygen species (ROS) accumulation. Their ineffectiveness in renal cell carcinoma indicates that ccRCC possesses the capacity to mitigate oxidative stress. Under physiological conditions, NRF2 activation protects renal epithelial cells by inducing cytoprotective genes in response to ROS. While this pathway exerts anti-inflammatory and tumor-suppressive effects during carcinogenesis, ccRCC co-opts NRF2 signaling to promote tumor growth, metastasis, and therapy resistance. This mechanism can also be exploited by ccRCC and papillary renal cell carcinoma (pRCC). Critically, NRF2 hyperactivation confers resistance to tyrosine kinase inhibitors (TKIs) including sunitinib [26], sorafenib [11, 27] and axitinib [28, 29], demonstrating its role in multidrug resistance. Furthermore, NRF2 modulates PD-L1 expression, potentially compromising immune checkpoint blockade efficacy [30–32]. Therefore, targeting the NRF2 pathway represents a promising strategy to overcome treatment resistance in ccRCC.

Mounting evidence indicates that aberrant activation of the NRF2/ARE pathway is a hallmark of RCC. While genomic alterations in core pathway components (*NFE2L2*, *KEAP1*, *CUL3*, *SIRT1*) occur in only 7.9% of papillary RCC (pRCC) and 3.2% of clear cell RCC (ccRCC) cases [33], epigenetic mechanisms such as KEAP1 promoter methylation also directly suppress its expression in ccRCC [34]. Critically, post-translational regulation represents the predominant mechanism for NRF2 stabilization and downstream target activation in RCC. Herein, we identify TSC22D4 as a novel disruptor of KEAP1-mediated NRF2 degradation. This TSC22D4-KEAP1 interaction confers oxidative stress resistance, drives tumour progression, and promotes metastasis in RCC. Nevertheless, a comprehensive elucidation of NRF2 dysregulation requires further investigation into alternative activation mechanisms beyond canonical degradation control.

TSC22D4 has been reported to be induced by transforming growth factor beta (TGF-β) signaling, a pathway with diverse roles in carcinogenesis. While TGF-β suppresses early tumorigenesis through the induction of differentiation programs, it paradoxically promotes metastatic progression in advanced cancers [35]. In the present study, we might demonstrate a functional crosstalk between the TGF-β and NRF2 pathways mediated by TSC22D4. This axis promotes migration and invasion in RCC. However, further studies are required to delineate the precise interplay between TGF-β and NRF2 signaling and to validate the clinical relevance of TSC22D4.

Extensive research has focused on developing NRF2 pathway inhibitors, with numerous natural and synthetic compounds reported to suppress NRF2 signaling through diverse mechanisms [36]. However, clinical translation remains limited by insufficient efficacy and off-target toxicity [37]. The identification of TSC22D4 as a novel upstream regulator of NRF2 unveils a druggable target within this pathway. The TSC22D4-KEAP1 interaction interface represents a novel site for developing next-generation inhibitors with improved specificity against NRF2-driven therapy resistance.

Ferroptosis plays a pivotal role in NRF2 pathway research. As a primary mediator of ferroptosis resistance, NRF2 critically influences renal cancer therapeutics. Targeted therapies, particularly TKIs such as sorafenib and sunitinib, form the cornerstone of renal carcinoma treatment. In recent years, the combination of immunotherapy and targeted therapy has played a pivotal role in the treatment of renal cell carcinoma. However, resistance to these therapies remains a major clinical challenge. Emerging evidence suggests that ferroptosis resistance is a critical contributor to resistance against both targeted agents and immunotherapy. As a regulator of ferroptosis through the modulation of SLC7A11, TSC22D4 may represent an important mediator of therapy resistance. Mechanistic investigation of TSC22D4 could therefore provide new insights into the molecular basis of resistance to combined targeted and immune therapies and may facilitate the development of more effective therapeutic strategies.

This study has several limitations. In particular, in the context of therapy resistance, we were unable to collect a sufficient number of clinical samples from patients with resistance to targeted therapy for validation. The limited sample size may introduce potential bias into our analyses, and therefore, the clinical relevance of some conclusions requires further validation in larger patient cohorts.

Our investigation of TSC22D4 reveals that its ferroptosis resistance contributes to sorafenib resistance in ccRCC, highlighting the potential of targeting the TSC22D4-NRF2-SLC7A11 axis to sensitize tumor cells to ferroptosis-inducing therapies. This mechanism establishes a novel therapeutic avenue for overcoming clear cell renal cell carcinoma.

## Materials and methods

### Cell culture and transfection

786-O, A498, and HEK-293T cell lines were obtained from the American Type Culture Collection (ATCC, Manassas, VA, USA). The cell lines were recently authenticated by short tandem repeat (STR) profiling and tested negative for mycoplasma contamination prior to use. The 786-O and A498 cells were maintained in RPMI-1640 medium supplemented with 10% fetal bovine serum (FBS) and 1% penicillin-streptomycin. HEK-293T cells were cultured in Dulbecco’s Modified Eagle’s Medium (DMEM; Gibco, Thermo Fisher Scientific) containing 10% FBS and 1% penicillin-streptomycin. All cell lines were incubated at 37 °C in a humidified atmosphere with 5% CO₂.

Transient transfection was performed using Lipofectamine RNAiMAX for small interfering RNA (siRNA) transfection or Lipofectamine 3000 for plasmid transfection (L3000015, Thermo Fisher Scientific), according to the manufacturer’s instructions. For transfection, cells were seeded in 24-well plates at a density of 2 × 10⁴ cells per well 24 h prior to transfection. Normally, a total of 0.5 μg of a certain plasmid dissolved in 50 μl serum-free medium was mixed with 1 μl Lipofectamine 3000 desolved into 50 μl serum-free medium for 5 min at room temperature. Cells were incubated with the transfection mixture for 6 h and then cultured in complete medium for 24 h to allow expression of the transfected gene.

### Construction of plasmids and site-directed mutagenesis

Human TSC22D4 and KEAP1 cDNAs were purchased from GeneChem (Shanghai, China) and subcloned into the pCMV-FLAG and pCMV-MYC expression vectors. In addition, TSC22D4 and KEAP1 were cloned into the pCDH-GFP-FLAG vector to generate GFP-tagged TSC22D4 and KEAP1 fusion constructs. Site-directed mutagenesis of TSC22D4 and KEAP1 was performed using the KOD-Plus Mutagenesis Kit (SMK-101, TOYOBO, Japan) according to the manufacturer’s instructions. All plasmid constructs were confirmed by Sanger sequencing.

### RNA interference and construction of stable cell lines

Negative control siRNA and specific siRNAs targeting TSC22D4 and KEAP1 were purchased from GenePharma (Shanghai, China). Transfection of siRNAs was performed according to the manufacturer’s instructions as previously described [38]. The sequences of the siRNAs are provided in the Supplementary Materials. We constructed stable cell lines following the protocols described previously [38]. Cells were selected with appropriate antibiotics to obtain stable clones.

### Immunoprecipitation and Western blotting

Transfected cells were harvested 24 h after transfection and lysed in BC100 lysis buffer supplemented with protease inhibitors on ice. Cell lysates were clarified by centrifugation at 12,000 × *g* for 15 min at 4 °C, and the supernatants were collected for immunoprecipitation. Equal amounts of protein were incubated overnight at 4 °C with monoclonal anti-FLAG antibody-conjugated M2 agarose beads (M8823, Millipore) with gentle rotation. The beads were then washed three times with FLAG lysis buffer and twice with BC100 buffer to remove nonspecifically bound proteins. Immunoprecipitated protein complexes were eluted by incubation with FLAG peptide (F4799, Sigma-Aldrich) according to the manufacturer’s instructions.

Western blot analysis was performed as previously described in our published study [38]. The primary antibodies used are as follows: anti-TSC22D4 mouse polyclonal antibody (SAB1412228, Sigma-Aldrich), anti-NRF2 rabbit monoclonal antibody (ab62352, Abcam), anti-KEAP1 mouse monoclonal antibody (60027-1-Ig, Proteintech), anti-β-actin mouse monoclonal antibody (ab179467, Abcam), anti-SLC7A11 rabbit monoclonal antibody (26864-1-AP, Proteintech), GAPDH mouse monoclonal antibody (60004-1-Ig, Proteintech) and Histone H3 rabbit polyclonal antibody (17168-1-AP, Proteintech). The secondary antibodies used were goat anti-rabbit IgG-HRP (sc-2004, Santa Cruz) and goat anti-mouse IgG-HRP (sc-2005, Santa Cruz).

### Quantitative RT-PCR

Quantitative RT-PCR was performed as previously described [38]. Total RNA was extracted by TRIzol reagent (15596026, Invitrogen), and cDNA was reverse transcribed using the High-Capacity cDNA Reverse Transcription Kit (4368813, ThermoFisher), according to the manufacturer’s instructions. qPCR was performed using the SYBR Green PCR Master Mix Kit (A46113, ThermoFisher). Endogenous β-actin was used for normalization.

### Cycloheximide chase assay

Cells (5 × 10⁵) were seeded per well in 6-well plates and allowed to adhere for 24 h. Cycloheximide (CHX; HY-12320, MedChemExpress) was added to the CHX-treated group at a final concentration of 100 μg/mL, while an equal volume of dimethyl sulfoxide (DMSO) was added to the control group. Cells were harvested at the indicated time points (0, 1, 2, 3, 4, and 5 h) by direct lysis in SDS-PAGE sample loading buffer containing 5% β-mercaptoethanol (CAS 60-24-2, Santa Cruz Biotechnology). Lysates were boiled for 10 min and subjected to Western blot analysis.

### Clone formation experiment

Cells (300 cells per well) were seeded into 6-well plates and cultured for approximately 2 weeks. When visible colonies had formed, the cells were fixed with 4% paraformaldehyde and stained with 0.1% bromophenol blue. Excess stain was gently removed by washing with phosphate-buffered saline (PBS) until colonies were clearly visible and countable. Images were captured, and the number and size of colonies in each well were quantified using consistent criteria.

### Scratch experiment

Cells were seeded in 6-well plates. Scratches were made by a 200 μL pipette tip, and PBS was used to wash away the removed cells and debris. Then, the cells were incubated with serum-free medium. Scratches were photographed at 0, 6 and 12 h post scratch. The width of gaps was measured by ImageJ (version 1.53j, National Institutes of Health, USA).

### Transwell experiment

For detailed descriptions of the migration and invasion assays, please refer to our previous article [38]. Briefly, 24-well plates with 0.4 μm pore Transwell inserts (Corning) were used. A total of 3 × 10⁴ cells, suspended in serum-free medium, were placed in the Transwell inserts, and the lower chambers were filled with 10% serum-containing medium. The plates were incubated for 24 h, and the inserts were then fixed with 4% paraformaldehyde for 20 min. After fixation, the inserts were washed with PBS and stained with 0.1% bromophenol blue. The non-migrating cells on the inner surface of the inserts were removed, and the inserts were imaged using a microscope.

For the invasion assay, Matrigel-coated inserts were used, and cells were seeded on top of the Matrigel. After 48 h, the inserts were collected, and the subsequent steps were the same as those for the migration assay.

### Ubiquitination experiments

Ubiquitination experiments were performed using immunoprecipitation and Western blotting techniques described before. Cells were treated with the proteasome inhibitor MG132 prior to lysis to prevent degradation of ubiquitinated proteins. The HA-tagged ubiquitin (Ub) plasmid was transfected into various stable cell lines. Protein A/G Magnetic beads (B23201, Bimake) were pre-incubated with an anti-NRF2 antibody at a dilution of 1:500 for 1 h at 4 °C to facilitate the specific binding of the antibody to the beads. After incubation, the beads were washed three times with cold PBS to remove any unbound antibodies. Endogenous NRF2 protein was subsequently isolated using Protein A/G Magnetic beads (B23201, Bimake) pre-incubated with an anti-NRF2 antibody. The beads were washed and boiled to release the NRF2 protein. The samples were then analyzed by Western blotting using an anti-HA antibody (66006-2-Ig, Proteintech).

### GSH, MDA, SOD, and CAT detection

Cells were seeded in 6-well plates at a density of 3 × 10⁴ cells per well and cultured under standard conditions (37 °C, 5% CO₂) for 24 h to achieve approximately 70–80% confluence. Cells were washed twice with cold PBS to remove any residual medium. The cells were harvested by scraping or trypsinization, followed by centrifugation at 1000 × *g* for 5 min to collect the cells. The cells were suspended in cold PBS and homogenized for a GSH assay kit (S0053, Beyotime, China), an MDA assay kit (S0131S, Beyotime, China), an SOD assay kit (S0109, Beyotime, China), and a CAT assay kit (S0051, Beyotime, China). All procedures were strictly followed by protocols.

### Lipid peroxidation detection and Flow cytometry analysis

Cells were seeded in 6-well plates at a density of 3 × 10⁴ cells per well and allowed to adhere for 24 h to reach approximately 70–80% confluence. The cells were incubated with C11-BODIPY™ 581/591 C11 (Invitrogen) at a final concentration of 1 μM in serum-free medium for 30 min at 37 °C, protected from light. After incubation, the cells were washed twice with pre-warmed PBS to remove any unbound probe. The cells were collected by trypsinization, followed by washing with cold PBS to prepare a single-cell suspension. For lipid ROS detection, the cells were analyzed by flow cytometry using a Beckman Coulter FC500 flow cytometer (Beckman Coulter, Inc.). Cells were excited with a 488 nm laser, and the emitted fluorescence was detected in 510 nm and 590 nm. The data were analyzed using FlowJo software (Tree Star, Inc.) to quantify the ROS levels based on the ratio of green to red fluorescence intensity.

### Immunohistochemical experiments

Immunohistochemical experiments were performed as previously described [38]. The protocol was strictly followed according to the instructions of the IHC kit (G1210-1, ThermoFisher). A light Olympus IX73 inverted microscope was used to observe the IHC slides and ImageJ was used to analyze the results. IHC scores were measured by the intensity of staining and the proportion of tumor cells. Positive reactions were defined as those showing brown signals in the cell cytoplasm. The intensity was scored: 0, negative; 1, weak; 2, moderate; 3, strong. The proportion of positive cells was scored: 0, less than 5%; 1, 5–25%; 2, 26–50%; 3, 51–75%; 4, greater than 75%. The final IHC score was calculated as the product of the intensity score and the proportion score.

### Immunofluorescence staining

For immunofluorescence staining, tissue sections were first fixed with 4% paraformaldehyde (PFA) in PBS at room temperature for 20 min. After fixation, sections were washed three times with PBS and permeabilized with 0.3% Triton X-100 in PBS for 15 min. Blocking was performed with 5% bovine serum albumin (BSA) in PBS for 1 hour at room temperature to prevent nonspecific binding. Primary antibodies were applied overnight at 4 °C. After incubation, sections were washed three times with PBS and incubated with appropriate secondary antibodies conjugated with fluorescent dyes for 1 h at room temperature. Secondary antibodies were Goat anti-Rabbit IgG secondary antibody (Cat # A-11011, Thermo Fisher Scientific) and Goat anti-Mouse IgG Secondary Antibody (Cat # A-11008, Thermo Fisher Scientific). For nuclear counterstaining, DAPI (1 μg/mL) (62248, Thermo Fisher Scientific) was applied for 5 min, followed by three washes with PBS. Sections were mounted with mounting medium containing an antifade reagent to prevent fluorescence quenching. Negative controls were performed by omitting the primary antibody or using an irrelevant isotype control antibody. Immunofluorescent images were captured using a FLUOVIEW FV3000 Confocal Microscope, and images were analyzed using ImageJ. Fluorescence intensity and colocalization were quantified by measuring the pixel intensity of specific regions of interest (ROI) within the images.

### Cell-derived xenograft model

For the cell-derived xenograft model, four-month-old female BALB/c nude mice were purchased from Cyagen Biosciences Inc. Renal cancer cells were harvested during the logarithmic growth phase, washed twice with sterile phosphate-buffered saline (PBS), and resuspended at an appropriate concentration. Each mouse was subcutaneously injected in the right flank with 1 × 10^6^ renal cancer cells in a total volume of 100 μL. For drug administration, tail vein injections were used. Tumor growth was monitored once per week using a digital caliper. Tumor volume was calculated according to the formula: V = (length × width²) / 2. Mice were randomly assigned to experimental groups after tumor establishment. Tumor measurements were performed in a blinded manner.

Throughout the experiment, mice were monitored regularly for body weight, general condition, and potential signs of toxicity. At the end of the treatment period, mice were sacrificed, and tumors were surgically excised, photographed, and weighed for further analysis. All animal experiments were approved by the Institutional Animal Care and Use Committee (IACUC) of Changzheng Hospital and conducted in accordance with institutional guidelines.

### Patient clinical information and tissue samples

Between March 2005 and December 2009, clinical information and tissue samples were obtained from 101 treatment-naïve patients with ccRCC at Fudan University Shanghai Cancer Center (FUSCC; Shanghai, China), all of whom underwent partial or radical nephrectomy. The exclusion criteria were as follows: (1) non-clear cell renal cell carcinoma on pathological review; (2) receipt of any antitumor treatment before surgery; (3) insufficient tumor tissue for immunohistochemical analysis; (4) incomplete clinicopathological information or missing follow-up data; and (5) coexistence of other malignant tumors. Detailed clinicopathological characteristics are provided in the Supplementary Table. Patient clinical data and expression were also obtained from public databases, including The Cancer Genome Atlas (TCGA, https://www.cancer.gov/ccg/research/genome-sequencing/tcga), the International Cancer Genome Consortium (ICGC, https://dcc.icgc.org/), and the Clinical Proteomic Tumor Analysis Consortium (CPTAC, https://hupo.org/Clinical-Proteome-Tumor-Analysis-Consortium-(CPTAC)). This study was approved by the Ethics Committee of Fudan University Shanghai Cancer Center, and informed consent was obtained from all patients.

### Statistics and data availability

All statistical and bioinformatic analyses were performed using R software (version 4.5.1) and GraphPad Prism 9 (version 9.2.0.332; GraphPad Software). Survival analysis was conducted using the Kaplan-Meier method, and differences between groups were evaluated using the log-rank test with the survival package (rdocumentation.org/packages/survival). Univariate and multivariate Cox proportional hazards regression analyses were performed to assess the independent prognostic value of TSC22D4. The analyses were performed within the RStudio integrated development environment (version 2025.09.1 + 401). Unless otherwise specified, comparisons between two groups were performed using a two-tailed Student’s *t* test, while one-tailed tests were applied only when a directional hypothesis was defined. *P* value < 0.05 was considered statistically significant. Sample sizes were chosen based on previous studies and common practice in the field rather than formal power calculations. For in vitro experiments, at least three independent experiments were performed. For in vivo studies, group sizes were determined based on feasibility and prior experience to ensure reproducibility. Homogeneity of variance was assessed using Levene’s test before applying parametric tests.

### Ethics approval and consent to participate

All methods used in this study were performed in accordance with the relevant guidelines and regulations. Informed consent was provided by all patients. All aspects of this study were approved by the Ethics Committee of Shanghai Changzheng Hospital. All animal procedures were performed in accordance with protocols approved by the Institutional Animal Care and Research Advisory Committee of Shanghai Changzheng Hospital.

## Supplementary information

Supplementary Methods and legends

Uncropped western blots

Supplementary Figure 1

Supplementary Table

## Data Availability

All data generated or analyzed during this study are available from the corresponding author upon reasonable request.
