# Supplementary material for: TSC22D4 drives clear cell renal cell carcinoma progression and therapy resistance by stabilizing NRF2 through KEAP1 disruption
Source: Cell Death Dis. 2026 Jun 5;17(1):702. doi: 10.1038/s41419-026-08950-4 (PMC13454145; doi:10.1038/s41419-026-08950-4)
Supplement: Supplementary file 1 [file 41419_2026_8950_MOESM1_ESM.docx]

**Supplementary Methods**

**In-gel Digestion**

For in-gel tryptic digestion, gel bands corresponding to the expected molecular weight of the target protein were carefully excised and destained using 50% acetonitrile in 50 mM ammonium bicarbonate (NH₄HCO₃). The gel pieces were subsequently dehydrated in 100% acetonitrile for 5 min, followed by reduction and alkylation with 10 mM TCEP and 40 mM CAA at 60 °C for 30 min. After incubation, the gel pieces were again dehydrated with 100% acetonitrile. The dried gel fragments were then rehydrated in 50 mM NH₄HCO₃ containing 2 μg trypsin and digested overnight at 37 °C. Following digestion, peptides were extracted using 50% acetonitrile supplemented with 0.1% formic acid. The extracted peptides were dried in a SpeedVacuum concentrator and subsequently reconstituted in 0.1% formic acid prior to LC-MS/MS analysis.

**LC-MS/MS Analysis**

Peptide samples were dissolved in mobile phase A (0.1% formic acid) and separated using an EASY-nLC 1200 system (Thermo Scientific). Chromatographic separation was performed at a constant flow rate of 400 nL/min with the following gradient: 2% to 7% mobile phase B (0.1% formic acid in 80% acetonitrile) over 1 min, 7% to 35% over 35 min, 35% to 55% over 9 min, increased to 100% within 7 min, and maintained at 100% for an additional 8 min. Eluted peptides were analyzed using a nano-electrospray ionization source coupled to a Q Exactive HF-X mass spectrometer (Thermo Scientific). The electrospray voltage was set to 2.0 kV. Full MS scans were acquired in the Orbitrap at a resolution of 60,000. The top 20 most abundant precursor ions were selected for MS/MS fragmentation using a normalized collision energy (NCE) of 27, and fragment ions were detected in the Orbitrap at a resolution of 15,000. Data were acquired in a data-dependent acquisition (DDA) mode with a dynamic exclusion time of 20 s.

**LC–MS/MS Data Processing**

Raw mass spectrometry data were processed and analyzed using Proteome Discoverer (version 2.4, Thermo Scientific) and searched against the human Swiss-Prot database (release 2023_09). Trypsin/P was specified as the proteolytic enzyme, allowing up to two missed cleavages. The precursor ion mass tolerance was set to 10 ppm, and the fragment ion mass tolerance was set to 0.02Da. Carbamidomethylation of cysteine was defined as a fixed modification, while oxidation of methionine and acetylation at the protein N-terminus were set as variable modifications. Peptide-spectrum matches were filtered with a high confidence threshold.

**Dual-luciferase reporter assay**

To evaluate NRF2 transcriptional activity, an antioxidant response element (ARE)-driven luciferase reporter system was constructed. Cells were co-transfected with the ARE firefly luciferase reporter plasmid and a Renilla luciferase plasmid as an internal control. After transfection for 24h, cells were lysed and luciferase activities were measured using a dual-luciferase reporter assay system (RG027, Beyotime) according to the manufacturer’s instructions. Firefly luciferase activity was normalized to Renilla luciferase activity to control for transfection efficiency. Each experiment was performed for five times, and data are presented as mean ± SD.

In details, cells were lysed using passive lysis buffer (PLB) according to the manufacturer’s instructions. The lysates were then centrifuged at 10,000×g for 3 minutes. The firefly luciferase assay reagent and Renilla luciferase assay buffer were equilibrated to room temperature before use. The Renilla luciferase substrate (100×) was kept on ice and diluted at a ratio of 1:100 in the Renilla luciferase assay buffer to prepare the working solution. Luciferase activity was measured using a luminometer according to the manufacturer’s instructions. The measurement interval was set to 2 seconds, and the integration time was set to 10 seconds, or adjusted as required by the instrument. Subsequently, 100 μL of firefly luciferase assay reagent was added to each sample, mixed gently, and the luminescence signal (relative light units, RLU) was measured. Following this, 100 μL of Renilla luciferase working solution was added to the same sample, mixed thoroughly, and the Renilla luciferase activity was recorded.

Relative luciferase activity was calculated by normalizing firefly luciferase activity to Renilla luciferase activity (Firefly/Renilla). The normalized values were used to compare the relative activation of the reporter gene among different experimental groups.

**Renal cancer organoid culture**

Renal cancer organoid was established using a commercially available human renal carcinoma organoid culture kit according to the manufacturer’s instructions (HY-K6126, MedChemExpress), with minor modifications. In short, fresh tumor tissues were immediately immersed in pre-cooled tissue preservation solution (HY-K6010, MedChemExpress) and temporarily stored at 4°C prior to processing. Under sterile conditions, tumor samples were washed with renal carcinoma organoid basal medium A or PBS to remove non-epithelial components, including adipose and muscle tissues. The tissues were then minced into small fragments (2 mm in diameter) and transferred into 15 mL conical tubes. An appropriate volume of tumor tissue digestion solution (HY-K6011, MedChemExpress) was added. Samples were incubated at 37°C for 0.5 h with gentle agitation until most tissue fragments could be aspirated using a 1 mL pipette. To terminate digestion, fetal bovine serum (FBS) was added to a final concentration of 2%, and the suspension was filtered through a 100 μm cell strainer on ice. The filtrate was centrifuged at 4°C, 250 × g for 3 min to collect cell pellets. The cell pellet was washed 3 times with renal carcinoma organoid basal medium A by centrifugation at 4°C, 250 × g for 3 min and subsequently used for organoid culture. Isolated tumor cells were resuspended in Matrigel (MCE) on ice at a density of approximately 4 × 10⁴ cells per 100 μL matrix. Using pre-wetted pipette tips, 25 μL of the cell–Matrigel mixture was seeded into each well of a 24-well plate, taking care to avoid bubble formation. Plates were incubated at 37°C with 5% CO₂ for 30 min to allow gel polymerization. After solidification, 500 μL of renal carcinoma organoid complete medium was gently added along the well edge to avoid disrupting the Matrigel dome. Organoids were cultured at 37°C in a humidified incubator with 5% CO₂. Medium was replaced every 3–4 days with pre-warmed complete medium. Renal cancer organoid formation was typically observed within 7–10 days.

Renal cancer organoid was incubated with TSC22D4-knockdown lentiviral particles in the presence of polybrene. After infection, organoids were re-embedded in ECM and cultured in complete organoid medium. Antibiotic selection was initiated 48–72 h post-infection to establish stable organoid lines. Gene expression was validated by qRT-PCR and western blotting. Tumor tissues were obtained with informed consent and approved by the institutional ethics committee.

**Supplementary Figure legends**

**Supplementary Figure 1A. Identification of KEAP1-interacting proteins by mass spectrometry**

Venn diagram showing the overlap of proteins identified in KEAP1 and IgG control groups. Representative proteins specifically enriched in the KEAP1 group are indicated.

**Supplementary Figure 1B. Expression of TSC22D4 in renal cancer cell lines**

Protein expression of TSC22D4 in renal cancer cell lines. TSC22D4 levels were assessed by Western blot analysis in human embryonic kidney cell line 293T and renal cancer cell lines OS-RC-2, 786-O, ACHN, A498, and 769-P. β-actin was used as the loading control. Representative images from three independent experiments are shown.

**Supplementary Figure 1C. Measurement of NRF2 transcriptional activity by dual-luciferase reporter assay**

NRF2 transcriptional activity was assessed using an ARE-related dual-luciferase reporter assay. Cells were co-transfected with an ARE luciferase reporter plasmid and a Renilla luciferase plasmid as an internal control. Relative luciferase activity was determined by normalizing firefly luciferase activity to Renilla luciferase activity. Relative luciferase activity was measured in 786-O and A498 cells expressing control, TSC22D4-sh1, or TSC22D4-sh2. Data are presented as mean ± SD from five replicates. Statistical significance was analyzed using Student’s t-test, with *P < 0.05 considered statistically significant.

**Supplementary Figure 1D. Kaplan–Meier survival curves of the FUSCC cohort**

Kaplan–Meier survival analysis of patients in the FUSCC cohort stratified by TSC22D4 expression. Overall survival was generated to evaluate the association between TSC22D4 expression and patient prognosis. overall survival was compared using the Kaplan–Meier method. Statistical significance was determined by the log-rank test. No significant survival difference was observed between the high- and low-TSC22D4 expression groups in this cohort.

**Supplementary Figure 1E-F. TSC22D4 knockdown enhances the response of renal cancer organoids to sorafenib**

Representative images of organoids derived from renal cancer cells stably expressing shControl, TSC22D4-sh1, and TSC22D4-sh2, following DMSO or sorafenib treatment. Renal cancer cells were cultured in a three-dimensional organoid system and treated with sorafenib under the indicated conditions. Representative bright-field images are shown. Quantitative analysis of organoid size was performed to evaluate the effect of TSC22D4 knockdown on sorafenib sensitivity. Data are presented as mean ± SD from three independent experiments. Statistical significance was determined using Student’s t-test, with *P < 0.05 considered statistically significant.

**Supplementary Figure 1G. Quantification of colony formation in A498 cells under different TSC22D4 conditions.**

Colony formation ability of A498 cells stably expressing empty vector (EV), wild-type TSC22D4, ETGE-mutant TSC22D4, shControl, TSC22D4-sh1, or TSC22D4-sh2 under sorafenib treatment was quantified. Data are presented as mean ± SD from three independent experiments.

**Supplementary Table legends**

**Table 1. Clinicopathological characteristics of patients in the FUSCC cohort.**

**Table 2. List of inhibitors used in this study.**

**Table 3. List of Primer sequences used in this study.**

**Table 4. Candidate proteins identified by mass spectrometry analysis shown in the Venn diagram.**
