# Supplementary material for: TSC22D4 drives clear cell renal cell carcinoma progression and therapy resistance by stabilizing NRF2 through KEAP1 disruption
Source: Cell Death Dis. 2026 Jun 5;17(1):702. doi: 10.1038/s41419-026-08950-4 (PMC13454145; doi:10.1038/s41419-026-08950-4)
Supplement: Supplementary file 2 [file 41419_2026_8950_MOESM2_ESM.docx]

Figure 1

Figure 2

Figure 3, 6 and supplementary Figure 1
